# MOCSE Centered on Students: Validation of Learning Demands and Teacher Support Scales

**DOI:** 10.3389/fpsyg.2020.582926

**Published:** 2020-10-02

**Authors:** Fernando Doménech-Betoret, Amparo Gómez-Artiga, Laura Abellán-Roselló, Esperanza Rocabert-Beút

**Affiliations:** ^1^Developmental and Educational Psychology, Universitat Jaume I, Castellón de la Plana, Spain; ^2^Developmental and Educational Psychology, University of Valencia, Valencia, Spain

**Keywords:** educational model, teacher support, learning demands, student motivation, questionnaire validation

## Abstract

Based on The Educational Situation Quality Model (MOCSE, acronym in Spanish) framework, the primary objective of this study is to test the factorial validity and reliability of two MOCSE measure instruments referred to the preactional-decisional phase, specifically to learning demands and teacher supports perceived by students to overcome such demands in the classroom context. The participants were 357 Spanish undergraduate students. The data obtained by exploratory and confirmatory factor analyses revealed that the “Learning Demands Scale” (MOCSE-LDS) has a two-factor structure: perceived desirability and feasibility of demands. The data also revealed that the “Teacher Support Questionnaire” (MOCSE-TSQ) is composed of ten independent factors or subscales with good psychometric validity and reliability properties. Finally, the Student’s *t*-test generally indicated that the constructs considered in the instruments did not differ in gender terms. In short, the results obtained for the validity and reliability of the two tested instruments were good. Thus, the application of instruments MOCSE-LDS and MOCSE-TSQ is satisfactorily supported by empirical data. The resulting scales can be useful for researchers and teachers. On the one hand, this study provides researchers with two valid and reliable tools that may contribute to investigate students’ motivation in the university classroom context based on MOCSE postulates. On the other hand, the two tested instruments may provide teachers and school psychologists with important information to implement preventive or intervention actions to improve students’ intention to learn. Teachers may also use them to evaluate their own teaching and to research their own classrooms. The implications for education according to MOCSE postulates are discussed.

## Introduction

The Educational Situation Quality Model (MOCSE, acronym in Spanish), devised by Doménech-Betoret (2006; 2013; 2018), is an instructional-motivational model that explains the functioning of an educational setting by organizing and relating the most important variables which, according to the literature, contribute to explain learning outcomes. Specifically, the model integrates motivational constructs and approaches from relevant psychological theories such as: *Job Demands-Resources Model* (JD-R) (Demerouti et al., 2001; Bakker and Demerouti, 2007, 2008), *Expectancy-Value Theory* (Eccles and Wigfield, 2002; Eccles, 2009), *Achievement goal theory* (Dweck and Leggett, 1988; Nicholls, 1989; Ames, 1992; Wigfield and Eccles, 2000), and *Theory of Action Control* (Heckhausen and Kuhl, 1985; Kuhl and Beckmann, 1985). It offers researchers a new tool to study how an educational setting operates and provides the teacher with a methodological procedure to guide and improve teacher practice. The model is made up of four sequential stages: (1) Student cognitive evaluation (learning demands and supports); (2) Intention to learn activation; (3) Action plan and teaching-learning process; (4) Learning outcomes. The stages are distributed into three broad phases: Preactional-decisional phase (Phase I), Actional phase (Phase II), Reflectional phase (Phase III).

**FIGURE 1:**
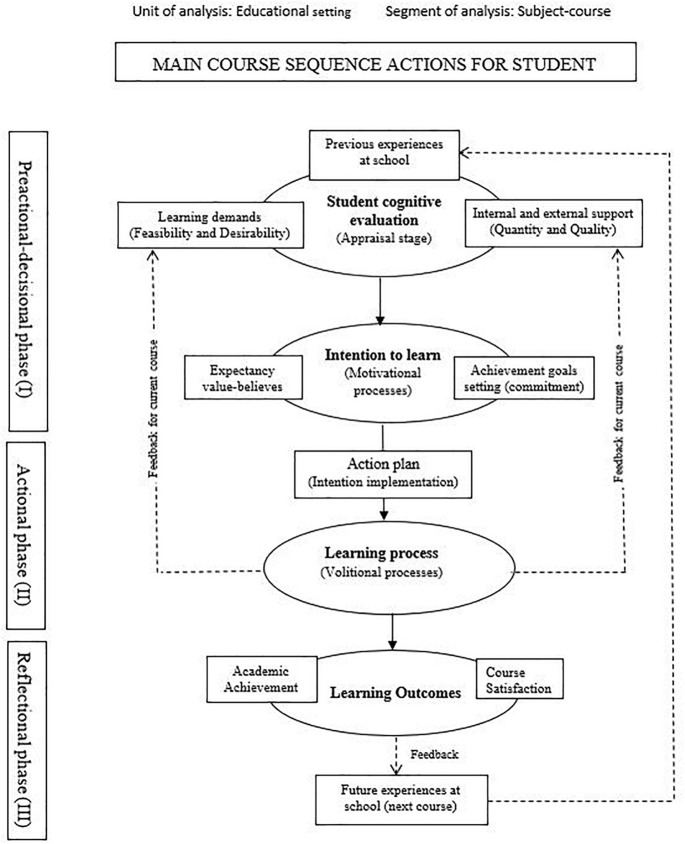
MOCSE diagram: Main course sequence actions for students. ^1^Connected with students’ interests, needs, and academic level (meaningful demands, Doménech-Betoret, 2018).

The model starts from a basic premise, which claims that “learning” requires students’ intention to learn to be activated at the beginning of the educational process, and it has to remain active until the process ends. Students’ intention to learn is generated or activated on the first days of the teaching-learning process according to the information they receive from the environment in terms of the demands required and the supports/resources received. However as the course unfolds, students’ perceptions are continuously updated and changing as a result of the constant (re)appraisals made by them (Doménech-Betoret, 2018; Doménech-Betoret et al., 2019). The model centered on students is displayed in Figure 1. See Doménech-Betoret (2018) for a profounder understanding of the model.

As seen in Figure 1, students’ perceptions of learning demands, and the supports they are provided with to overcome such demands (Stage 1), are assumed to predict intention to learn (Stage 2) which, in turn, affects the role adopted by students in classrooms (e.g., active-passive, engagement-disengagement, etc.) (Stage 3) which, in turn, finally has an effect on learning outcomes, such as academic achievement and course satisfaction (Stage 4). The whole model pivots around intention to learn (Stage 2), where the components from Stage I, such as demands and supports, are considered antecedents or predictive variables, whereas those from Stages 3 and 4 are taken as consequences or outcome variables. The temporal dimension of the MOCSE model is based on the Theory of Action Control (Heckhausen and Kuhl, 1985; Kuhl and Beckmann, 1985) as we explain below.

In the present study, we paid attention to Phase I (Preactional-decisional phase), specifically to the learning demands and supports (Stage 1) perceived by students, which are assumed to predict intention to learn (Stage 2) when motivational processes are involved. During the first process, initial wishes, desires and hopes are evaluated in terms of their chances of being fulfilled and transformed into personal goals (commitment) (Doménech-Betoret, 2018).

### Contribution of the Job Demands and Resources Model (JD-R) and the Expectancy-Value Theory to Explain the Preactional-Decisional Phase of MOCSE

According to the MOCSE Model, before making the decision to assume and commit to achieve (or not) the learning objectives (goals) planned in a specific subject matter, students follow several cognitive processes that can be explained by the JD-R Model (Evaluation Process) and Expectancy-Value Theory (Anticipatory cognitive process). Accordingly, and based on the MOCSE postulates, this phase predetermines, from the beginning of the course, the degree of student involvement in the teaching-learning process.

According to the Job Demands-Resources (JD-R) Model (Demerouti et al., 2001; Bakker and Demerouti, 2007, 2008), burnout and work engagement are two psychological states that play a key role in the workplace, and job demands and job resources/supports evoke two underlying psychological processes: (a) an energetic process during which high job demands lead to burnout and, in turn, they affect health; (b) a motivational process during which job resources promote engagement and, in turn, organizational commitment, but burnout also increases when job resources are lacking (Schaufeli and Bakker, 2004). The JD-R Model was traditionally utilized in the job context. The MOCSE model applies this theory to the school context.

Intention to learn (Stage 2) is a complex construct in which multiple variables are involved. For us, intention to learn has the same meaning as motivation to learn. Intention is considered the immediate antecedent of action (Doménech-Betoret, 2018). Intention to learn is basically made up of the anticipatory cognitive motivators proposed by the Expectancy-Value Theory. Authors from this tradition, such as Pintrich and de Groot (1990), distinguish three general categories of motivational constructs that are relevant for motivation in educational contexts: (a) individual perceptions and beliefs about the ability to perform a task/subject (e.g., expectancy of success, expectancy of control, etc.), Will I be able to pass this subject?; (b) the reasons or intentions to get involved in a task/subject (e.g., goals, value of the subject, etc.), Why am I going to get involved in this subject?; (c) affective reactions to a task/subject (e.g., expectations of enjoyment, feeling stressed, etc.). How will I feel in this subject during the course?

In the Preactional-decisional phase (I), MOCSE attempts to bridge both theories (JD-R and Expectancy-Value Theory) to explain how students make decisions to be involved in the teaching-learning process of a specific subject. Accordingly, and based on prior research (Patrick et al., 2007; Wentzel, 2009; Lin-Siegler et al., 2016), the model starts with a basic premise: “the perception that each student forms of; first, the scheduled learning demands that they must complete to pass a specific subject; second, the support that they perceive as being provided, mainly from the teacher and family, to face these demands, is crucial to activate students’ intention to learn” (Doménech-Betoret et al., 2019, p. 3). Moreover, MOCSE, in line with the JD-R Model, defends that demands are probably more related to affective reactions, whereas supports (from teacher, peers, family, etc.) are probably more related to expectancy beliefs (expectancy of success, expectancy of control, etc.).

Regarding learning demands, the “Model of Action Phases” (Heckhausen and Gollwitzer, 1987), based on the Theory of Action Control (Heckhausen and Kuhl, 1985; Kuhl and Beckmann, 1985), postulates that the person’s decision to set a goal intention is commonly assumed to depend on both the desirability and feasibility (Fishbein and Ajzen, 2010) of demands. Using the same reasoning, in the classroom context it also depends on both the desirability and feasibility of the academic-learning demands planned for a specific subject. In the classroom context, students’ subjective evaluation of both desirability and feasibility will be better insofar as demands connect with students’ interests, needs and academic level (meaningful demands, see Doménech-Betoret, 2018).

Regarding teaching support, prior research has shown that positive students’ perceptions of emotional/affective and instrumental/academic teacher support are positively related to students’ intrinsic motivation and positive emotional responses (Katz et al., 2009; Roorda et al., 2011; Federici and Skaalvik, 2014a). In the same vein, a supportive student-teacher relationship is particularly relevant for student motivation (Roorda et al., 2011; Dietrich et al., 2015). Finally, based on the Social Cognitive Theory (Bandura, 1986), Federici and Skaalvik (2014b) argue that teachers who provide both emotional/affective and instructional/academic support likely persuade students to believe in their ability and, as a consequence, to exert more effort to complete and master their learning activities.

In short, the above empirical findings seem to indicate a clear positive association between JD-R Model (demands and supports) and Intention to learn in the classroom context in accordance with the MOCSE postulates.

### Demands and Supports for Students in the Classroom Context

In the JD-R Model context, job demands are defined as “physical, psychological, social or organizational aspects of work that require physical and/or psychological effort (cognitive or emotional), and are associated with a certain physiological and/or psychological cost” (Bakker and Demerouti, 2007, p. 312). Job resources/supports refer “to the physical, psychological, social, or organizational aspects of the job that may reduce job demands and the associated physiological and psychological cost, are functional for achieving work goals, and stimulate personal growth, learning and development” (Hakanen et al., 2006, p. 497).

Applying this theory to the school context first requires a thorough analysis of what kind of learning demands related to a specific subject matter must students assume and, second, what kind of support (from teacher, family, etc.) should students be provided with during the curse to complete these demands. According to Lin-Siegler et al. (2016), students’ beliefs about themselves and their environment influence their motivation. Previous research based on the Job Demands-Resources Model (JD-R) (Demerouti et al., 2001) suggests that students’ perceptions of the resources/supports they are provided with (by teacher, family, etc.) to complete learning demands may have strongly influenced the variables related to intention to learn (motivational processes), such as expectancy-value believes (Abellán-Roselló, 2016) and goal pursuit (Wentzel et al., 2010; King and McInerney, 2014).

### Learning Demands

Learning demands refer to a specific subject in the classroom context, and basically to the tasks that students have to complete (procedural demands) and the contents they have to study (conceptual demands) to fulfill the programmed objectives and pass the subject (Doménech-Betoret et al., 2019). Students are expected to assume and pursue the learning objectives/goals planned by the teacher to fulfill them.

The concept of intention is central to theorizing on human goal striving. “In traditional theories on goal striving, the intention to achieve a certain goal is seen as an immediate determinant of goal achievement” (Brandstätter et al., 2001, p. 946). Accordingly, for decades, research was centered to identify the factors that determine the formation of strong intentions (e.g., Atkinson, 1957; Fishbein and Ajzen, 1975; Ajzen and Fishbein, 1980; Heckhausen et al., 1985). The Theory of Action Control (Heckhausen and Kuhl, 1985; Kuhl and Beckmann, 1985) extended this concern to the gap between intention and action. Integrating motivational and volitional (Executive motivation) aspects have contributed to a more comprehensive representation of the learning process. This theory promotes a new approach centered on the temporal dimension of motivation.

Based on the Theory of Action Control (Heckhausen and Kuhl, 1985; Kuhl and Beckmann, 1985), the “Model of Action Phases” (Heckhausen and Gollwitzer, 1987) applies psychological theories about cognition and motivation to investigate the processes that occur during goal pursuit, from setting a goal to fulfilling it. It offers a time perspective on goal striving and thus takes an integrative view by examining both goal setting and goal implementation within a single conceptual framework. According to this model, goal pursuit is carried out in four successive action phases: the predecisional, preactional, actional and postactional phases (for a summary, see Gollwitzer, 1990). The first stage of the motivated behavioral process (predecisional) is to choose among competing wishes and turning them into binding goals (goal intentions). Usually, the variables related to expectancy-value theories are employed to explain the forming of a goal intention. Forming a goal intention is the first step to fulfill a certain desired outcome. During this process, people deliberate (deliberative mindset) and carry out an analysis of a goal’s *feasibility* and *desirability* (Gollwitzer, 1990; Gollwitzer and Bayer, 1999). In short, the decision to set a goal intention is commonly assumed to depend on both desirability and feasibility (Fishbein and Ajzen, 2010). That is, goals are most likely to be set when the anticipated endstate is subjectively evaluated as both desirable (I want X!) and feasible (Is X affordable for me?). From a psychological perspective, “a strong desire to attain a goal is not sufficient for the formation of a goal intention; in addition, one must be confident that the chances of attaining the goal are high” (Doménech-Betoret et al., 2019, p. 4). In the classroom context, students’ subjective evaluation of both desirability and feasibility will be better insofar as the teacher adapts learning demands to students’ personal characteristics; that is, to what extent demands connect with students’ interests, needs and academic level (meaningful demands, see Doménech-Betoret, 2018).

### Teacher-External Support Resources to Complete the Required Learning Demands

According to the self-determination view, teacher support occurs when students perceive cognitive, emotional or autonomy-oriented support from a teacher during their learning process. According to the broad perspective of the social support model, based on Tardy’s (1985) teacher support is defined as a teacher giving informational, instrumental, emotional, or appraisal support to students in any environment. As we can see, there is lack of consistency in the definition and terminology used with the supports provided by teachers, but most authors usually distinguish between instructional-instrumental and affective-social supports (Doménech-Betoret, 2018). Instructional-instrumental support provided by teachers aims to help students’ content domain and to achieve learning demands. The affective-social support provided by teachers aims to meet students’ psychological needs and wishes in the classroom context. Instructional-instrumental support is related to academic dimension, whereas affective-social support is related to intrapersonal or interpersonal dimensions. Teacher affective-social support enhances a teacher’s relationship with students. Teachers who show care and concern for their students receive the same treatment from students by, for instance, adhering to classroom norms (Chiu and Chow, 2011). In the tested instrument, we considered both instructional-instrumental and affective-social supports (see Table 1).

**TABLE 1:** Characteristics of the considered teacher support variables.

Considered supports (alphabetical order)	Type of support^a^	Classroom’s dimension affected^b^
Acknowledging the student’s effort	Affective-social	Intrapersonal
Autonomy support	Instructional-instrumental	Intrapersonal
Awakening interest in the subject	Affective-social	Intrapersonal
Content comprehension support	Instructional-instrumental	Academic
Guiding students in their learning	Instructional-instrumental	Academic
Peer support	Affective-social	Interpersonal
Providing didactic resources	Instructional-instrumental	Academic
Providing feedback	Instructional-instrumental	Academic
Relatedness support	Affective-social	Interpersonal
Self-competency support	Affective-social	Intrapersonal

*^*a*^Type of support: Instructional-instrumental versus Affective-social. ^*b*^Classroom dimension: Academic, Interpersonal, Intrapersonal.*

## Materials and Methods

### Participants and Procedure

The sample consisted of 357 Spanish undergraduate students, of whom 56 were male (15.7%), and 301 were female (84.3%). They were aged between 19 and 50 years (*M* = 22.17, *SD* = 4.22). The participants studied the Educational Psychology and Education degree at two Universities located in east Spain. The participants completed an online version of the two measure instruments during a class session. Participation in the study was completely voluntary. Confidentiality and personal data protection were guaranteed in accordance with current Spanish laws.

### Measures

The last versions of two self-report measure instruments tested for validation in the current study are described below. Both instruments were originally constructed in accordance with the MOCSE principles and theoretical directions. They have been periodically revised and refined over time based on previous research conducted in the university context (Doménech-Betoret, 2006, 2012; Doménech-Betoret et al., 2014; Doménech-Betoret, 2018). Students completed an online version of both instruments in class.

#### Learning Demands Scale (MOCSE-LDS)

This scale comprises 17 items and was devised to measure students’ perception of learning demands in a specific subject matter in desirability and feasibility terms. Students indicated their level of agreement on a Likert response scale ranging from 1 (totally agree) to 6 (totally disagree).

#### External Support: Teacher Support Questionnaire (MOCSE-TSQ)

This self-report questionnaire comprises 72 items distributed on 10 scales. It was devised to measure students’ perception of teaching support in a specific subject matter to help students to achieve learning demands. Students indicated their level of agreement on a Likert response scale ranging from 1 (totally agree) to 6 (totally disagree).

Finally, an additional shortened questionnaire to measure intention to learn components was used to explore the relations between demands-supports variables (from Stage I) and intention to learn components (from Stage 2). See Doménech-Betoret et al. (2019) to get the complete questionnaire.

– The Intention to Learn Questionnaire (MOCSE-ILQ). It is made up of two basic dimensions:
  (I) Expectancy Beliefs. This scale measures students’ anticipatory cognitive responses and emotional reactions. It is composed of two factors: 1.1. Expectancy of Success and 1.2. Process Expectancy. The first factor (Expectancy of Success) is made up of 10 items (α = 0.92). Response scale: completely disagree (1) to completely agree (6). This construct comprises both self-efficacy expectancy and outcome expectancy (Liem et al., 2008). The second factor (Process Expectancy) is made up of 10 items (α = 0.96). Response scale: quite unlikely (1) to quite likely (6). This construct refers to the affective reactions that students expect to experience in their interaction with the teacher and subject during the course.
  (II) Achievement Goals. It measures the achievement goal setting by students according to the degree of commitment that they are willing to make regarding learning demands. Response scale: Completely disagree (1) to Completely agree (6). It is composed of two factors: 2.1. Mastery goals and 2.2. Avoidance goals. The first factor (Mastery goals) is made up of five items (α = 0.96). Students who adopt a mastery goal focus on improving their competence and progress in an academic task/subject. The second factor (Avoidance goals) is made up of four items (α = 0.91). Students who adopt avoidance goals make the minimum effort, or even try to avoid learning, and work avoidance represent the absence of an achievement goal (Elliot, 1999).

### Data Analysis

First, an exploratory factor analysis (EFA) was conducted on each instrument (previous version) to examine the latent factor structure using SPSS package 25.00 (IBM Corp, 2018). An observed measure was obtained by averaging the items included in each factor or subscale. Second, a Confirmatory Factor analysis (CFA) was performed to confirm the factor structure obtained with the EFA. The goodness-of-fit statistics of the hypothesized model was computed using the EQS program (Bentler, 2006). Raw data were used as the data matrix. As the chi-square test is sensitive to sample size, using relative fit indices like CFI, the NNFI and RMSEA is highly recommended (Bentler, 1990). Values up to 0.08 for RMSEA indicate an acceptable fit, whereas values above 0.08 indicate a poor fit (Browne and Cudeck, 1993). For NNFI and CFI, values above 0.90 (Hoyle, 1995), or even 0.95 (Hu and Bentler, 1999), were fixed as the cut-off point. Third, Cronbach’ α for examining the instruments’ reliability was calculated. Finally, gender differences were examined with the Student’s *t*-test for independent samples.

## Results

### Learning Demands Scale (MOCSE-LDS)

#### Exploratory Factor Analysis

An EFA (principal component method with varimax rotation) was conducted on the 17-item scale to examine the latent factor structure. Two factors, regarding the desirability and feasibility concepts with learning goals, were extracted (eigenvalue > 1). They accounted for 67.374% of total variance. Factor 1 (Desirability) was made up of 11 items and accounted for 42.19% of variance. Factor 2 (Feasibility) was made up of six items and accounted for 25.18% of variance.

#### Confirmatory Factor Analysis

The two-factor structure scale obtained with the EFA was tested to carry out the CFA. The scale factor structure was optimized when six items were removed following the recommendations of the Wald and Lagrange test in the EQS program. Then the 11-item scale was tested again. As multivariate kurtosis (Mardias’s coefficient = 29.5452, normalized estimate = 16.4816) indicated that normal distribution was violated, the ML robust method of estimation was used. The obtained fit indices (χ*^2^* = 104.662; *p* = 0.0000, d.f. = 43; NNFI = 0.971; CFI = 0.977; RMSEA = 0.064) showed a good data fit for the 11-item two-factor structure scale. See Table 2 for more details.

**TABLE 2:** MOCSE-LDS factor structure, items’ standardized coefficients and their contribution to the corresponding factor (*R*-squared).

Factor 1: Desirability [Demandas atractivas] (α = 0.939) Mean (*SD*) = 1.93 (0.64)		St. Coef.	*R*^2^
1.	*Los contenidos que tendré que estudiar en esta asignatura parecen interesantes.*	0.944F1	0.892
	The contents I will have to study in this subject seem interesting.		
2.	*Las actividades y tareas que ha planteado el profesor/a en esta asignatura han despertado mi curiosidad.*	0.806F1	0.650
	The activities and tasks that the teacher considers in this subject the teacher have aroused my curiosity.		
3.	*Los contenidos que tendré que estudiar en esta asignatura han despertado mi curiosidad.*	0.929F1	0.863
	The contents I will have to study in this subject have aroused my curiosity.		
4.	*Las actividades y tareas que se me solicitan para superar esta asignatura conectan con mis necesidades personales y/o profesionales.*	0.735F1	0.541
	The activities and tasks that I am asked in order to pass this subject are related to my personal and/or professional needs.		
5.	*Los contenidos que tendré que estudiar en esta asignatura parecen atractivos.*	0.923F1	0.853
	The contents I will have to study in this subject seem appealing.		

**Factor 2: Feasibility [Factibilidad] (α = 0.916) Mean (*SD*) = 3.90 (1.20)**			

6.	*El nivel de exigencia establecido para superar la parte teórica es muy alto.*	0.860F2	0.740
	The set level of demand to pass the theoretical part is very high.		
7.	*El esfuerzo que hay que hacer para sacar una buena nota es excesivo.*	0.828F2	0.686
	The effort that has to be made to obtain a good mark is too much.		
8.	*El nivel de exigencia establecido para superar la parte práctica es muy alto.*	0.805F2	0.648
	The set level of demand to pass the practical part is very high.		
9.	*El volumen de trabajo que se pide para superar esta materia es excesivo.*	0.789F2	0.622
	The requested workload to pass this subject matter is too much.		
10.	*Los criterios de evaluación que el profesor/a ha establecido para superar esta asignatura me parecen demasiado exigentes.*	0.791F2	0.625
	I think that the evaluation criteria that the teacher has set to pass this subject are too demanding.		
11.	*Los objetivos que se pretenden alcanzar en esta materia son demasiado exigentes.*	0.747F2	0.558
	The objectives to be fulfilled in this subject matter are too demanding.		

**St. Coef., Standardized coefficient.**

#### Reliability Analysis Findings as a Result of the CFA

The Cronbach’s alpha internal consistency coefficient for the total questionnaire and each scale in the instrument was calculated. For the total questionnaire (MOCSE-LDS), the alpha coefficient was α = 0.772 Cronbach’s alpha values for these two scales are: α = 0.939 for Factor 1 (Desirability) and α = 0.916 for Factor 2 (Feasibility).

### Teacher Support Questionnaire (MOCSE-TSQ)

#### Exploratory Factor Analysis

An EFA (principal component method with varimax rotation) was conducted on the total questionnaire composed of 72 items to examine the latent factor structure. A cross loading problem (>0.40, according to Stevens, 2002) was observed for 12 items, so they were removed and the EFA was repeated again with the remaining 60 items. Ten factors were extracted, with an eigenvalue higher than 1, which accounted for 74.04% of total variance. Factor 1 (Content comprehension support) was made up of 10 items, and accounted for 13.48% of variance; Factor 2 (Autonomy support) was made up of seven items and accounted for 8.10% of variance; Factor 3 (Relatedness support) was made up of 10 items and accounted for 8.03% of variance; Factor 4 (Peer support) was made of five items and accounted for 7.40% of variance; Factor 5 (Awakening interest in the subject) was made of five items and accounted for 6.89% of variance; Factor 6 (Acknowledging the student’s effort) was made of five items and accounted for 6.77% of variance; Factor 7 (Self-competency support) was made of five items and accounted for 6.58% of variance; Factor 8 (Guiding students in their learning) was made of five items and accounted for 6.54% of variance; Factor 9 (Providing didactic resources) was made of four items and accounted for 5.48% of variance; Factor 10 (Providing feedback) was made of four items and accounted for 4.73% of variance.

#### Confirmatory Factor Analysis

The 10-factor structure scale obtained with the EFA was tested to carry out a CFA. The CFA provides a regression coefficient and an error number showing the degree of relation between each item and its corresponding latent variable or factor. The scale factor structure was optimized when five items were removed following the recommendations of the Wald and Lagrange test in the EQS program. Then the whole scale made up of 55 items was tested again. As multivariate kurtosis (Mardias’s coefficient = 760.176, normalized estimate = 90.568) indicated that normal distribution was violated, the ML robust method of estimation was used. The obtained fit indices (χ*^2^* = 2,265.183; *p* = 0.0000, d.f. = 1385; NNFI = 0.929; CFI = 0.934; RMSEA = 0.042) indicated that the questionnaire factor structure, composed of 10 scales, satisfactorily fitted the data. See Table 3 for details.

**TABLE 3:** MOCSE-TSQ factor structure, items standardized coefficients and their contribution to the corresponding factor (*R-*squared).

Factor 1: Content comprehension support [Apoyo a la comprensión del contenido] (α = 0.941) Mean (*SD*) = 4.41 (1.06)		Stand. Coef.	*R*^2^
1.	*Desde el principio, las explicaciones del profesor han sido claras y comprensibles, y creo que serán así a lo largo del curso.*	0.905F1	0.818
	Since the beginning, the teacher’s explanations have been clear and understandable, and I think this will continue throughout the course.		
2.	*Desde el principio, las explicaciones del profesor/a se han podido seguir bien, y creo que será así a lo largo del curso.*	0.918F1	0.842
	Since the beginning, the teacher’s explanations have been followed well, and I think this will continue throughout the course.		
3.	*Desde el principio, las explicaciones del profesor/a han sido lógicas y bien organizadas, y creo que serán así a lo largo del curso.*	0.894F1	0.798
	Since the beginning, the teacher’s explanations have been logical and well organized, and I think this will continue throughout the course.		
4.	*Desde el principio, el profesor/a ha utilizado en sus explicaciones un lenguaje y ritmo adecuado para facilitar nuestra comprensión, y creo que seguirá así a lo largo del curso.*	0.806F1	0.649
	Since the beginning, the teacher has used a suitable language and pace to help us to understand his/her explanations, and I think this will continue throughout the course.		
5.	*Desde el principio, las explicaciones del profesor/a se han ajustado al nivel de los alumnos/as para que éstos las puedan entender, y creo que será así a lo largo del curso.*	0.838F1	0.702
	Since the beginning, the teacher’s explanations have adapted well to the students’ level so they can understand them, and I think this will continue throughout the course.		
6.	*Desde el principio, el profesor/a ha destacado las ideas clave o aspectos más relevantes de lo explicado, y creo que seguirá así a lo largo del curso.*	0.805F1	0.648
	Since the beginning, the teacher has emphasized key ideas or the most relevant aspects of what has been explained, and I think this will continue throughout the course.		
7.	*Desde el principio, el profesor/a ha hecho un esquema/guión de lo que iba a explicar y ha indicado claramente el paso de un punto a otro, y creo que seguirá así a lo largo del curso.* Since the beginning, the teacher has made an outline/script of what (s)he was going to explain and has	0.677F1	0.459
	clearly indicated the step to take from one point to another, and I think this will continue throughout the course.		

**Factor 2: Relatedness and Teacher accessibility [Accesibilidad y cercanía del professor] (α = 0.931) Mean (*SD*) = 4.92 (0.79)**			

8.	*Desde el principio, este profesor/a se ha preocupado por nuestro aprendizaje y siempre se ha mostrado predispuesto a ayudar, y creo que seguirá siendo así a lo largo del curso.*	0.889F2	0.790
	Since the beginning, the teacher has considered our learning and has always been willing to help, and I think this will continue throughout the course.		
9.	*Desde el principio, este profesor/a siempre se ha mostrado predispuesto a resolver nuestras dudas, y creo que seguirá siendo así a lo largo del curso.*	0.829F2	0.687
	Since the beginning, the teacher has always been willing to solve our doubts, and I think this will continue throughout the course.		
10.	*Desde el principio, este profesor/a siempre se ha mostrado predispuesto a orientarnos cuando nos han surgido dificultades en completar alguna tarea, y creo que seguirá siendo así a lo largo del curso.*	0.871F2	0.759
	Since the beginning, the teacher has always been willing to guide us when we had difficulties completing some task, and I think this will continue throughout the course.		
11.	*Desde el principio, este profesor/a ha resuelto con prontitud y eficacia las dudas que le hemos planteado los estudiantes, y creo que seguirá siendo así a lo largo del curso.*	0.868F2	0.754
	Since the beginning, the teacher has quickly and efficiently solved the students’ doubts, and I think this will continue throughout the course.		
12.	*Desde el principio, este profesor/a ha estado siempre accesible ya sea de forma presencial o a distancia, y creo que seguirá siendo así a lo largo del curso.* Since the beginning, the teacher has always been available either face-to-face or virtually, and I think this will continue throughout the course. Since the beginning, the teacher has treated us with respect, and I think this will continue throughout the course.	0.806F2	0.650
13.	*Desde el principio, el profesor/a nos ha tratado con respeto, y creo que seguirá siendo así a lo largo del curso.*	599F2	0.359
14.	*Desde el principio, el profesor/a se ha mostrado abierto y dialogante, y creo que seguirá siendo así a lo largo del curso.*	770F2	0.593
	Since the beginning, the teacher has been open and willing to talk, and I think this will continue throughout the course		
15.	*Desde el principio, el profesor/a se ha mostrado cercano, y creo que seguirá siendo así a lo largo del curso.*	0.724F2	0.524
	Since the beginning, the teacher has been close, and I think this will continue throughout the course.		

**Factor 3: Autonomy support [Apoyo a la autonomía] (α = 0.881) Mean (*SD*) = 4.01 (0.93)**			

16.	*Desde el principio, el profesor/a nos ha ofrecido la oportunidad de enfocar y organizar la realización de las tareas cómo deseemos.*	0.779F3	0.607
	Since the beginning, the teacher has given us the opportunity to focus on and organize tasks as we wish		
17.	*Desde el principio (siempre respetando el programa de la asignatura), el profesor/a nos ha incitado a que tomemos nuestras propias decisiones sobre cómo enfocar el trabajo y estudio de esta materia.*	0.781F3	0.610
	Since the beginning (by always respecting the subject syllabus), the teacher has encouraged us to make our own decisions about how to approach the work and the study of this subject matter.		
18.	*Desde el principio, este profesor/a nos ha dado la oportunidad de enfocar y organizar el trabajo de los temas como deseemos.*	0.862F3	0.742
	Since the beginning, this teacher has given us the opportunity to approach and organize the work in the subject matters as we wish		
19.	*Desde el principio, este profesor/a nos ha ofrecido la oportunidad de que podamos elegir entre un abanico de tareas, actividades, lecturas, etc., en función de nuestras preferencias.*	0.772F3	0.596
	Since the beginning, this teacher has given us the opportunity to choose from a range of tasks, activities, readings, etc., according to our preferences.		
20.	*Desde el principio, el profesor/a nos ha incitado a que hagamos autoevaluaciones de nuestro aprendizaje para tomar conciencia de nuestros aciertos y de nuestros errores.*	0.655F3	0.429
	Since the beginning, the teacher has encouraged us to self-assess our learning to be aware of our successes and our mistakes.		
21.	*Desde el principio, este profesor/a ha fomentado que trabajemos esta materia con cierta autonomía e independencia para que aprendamos a ser personas autónomas en el futuro.*	0.604F3	0.365
	Since the beginning, this teacher has prompted us to work this subject matter somewhat autonomously and independently so we can learn to be autonomous people in the future.		
22.	*Desde el principio, el profesor/a nos ha exigido reflexiones personales sobre las actividades y tareas encomendadas.*	0.582F3	0.338
	Since the beginning, this teacher has expected us to personally reflect on the entrusted activities and tasks.		

**Factor 4: Peer Support [Apoyo entre iguales] (α = 0.954) Mean (*SD*) = 4.48 (1.14)**			

23.	*Por lo que he observado, parece que habrá un buen clima de compañerismo en clase.*	0.919F4	0.845
	From what I have observed, there will be a good classmate climate in the classroom.		
24.	*Los compañeros de clase parecen abiertos y amigables.*	0.918F4	0.843
	My classmates seem open and friendly.		
25.	*Los compañeros/as de clase me han causado buena impresión desde el principio.*	0.896F4	0.804
	I have always had a good impression of my classmates since the beginning.		
26.	*Mis compañeros/as se muestran cercanos y dispuestos a ayudar.*	0.875F4	0.766
	My classmates seem close and willing to help.		
27.	*Los compañeros de clase transmiten confianza y “buen rollo.”*	0.885F4	0.784
	My classmates transmit trust and a good environment.		

**Factor 5: Awakening interest in the subject [Despertar el interés por la materia] (α = 0.941) Mean (*SD*) = 4.80 (0.91)**			

28.	*En la presentación de la asignatura el profesor/a ha explicado para qué nos va a servir aprender y dominar esta materia.*	876F5	0.767
	When presenting the subject, the teacher has explained what learning and mastering this subject will serve		
29.	*En la presentación de la asignatura el profesor/a nos ha explicado el por qué esta materia es importante.*	899F5	0.808
	When presenting the subject, the teacher explained to us why this subject is important.		
30.	*En la presentación de la asignatura el profesor/a ha explicado la utilidad que tiene esta materia para la vida real.*	0.897F5	0.805
	When presenting the subject, the teacher has explained this subject’s usefulness for real life.		
31.	*En la presentación de la asignatura, el profesor/a ha explicado con claridad la importancia que tiene esta materia para nuestra formación.*	0.876F5	0.767
	When presenting the subject, the teacher has clearly explained this subject’s importance for our training.		
32.	*Desde el principio, el profesor/a nos hizo ver la importancia y utilidad de esta asignatura.*	0.782F5	0.612
	Since the beginning, the teacher has allowed us to see this subject’s importance and usefulness.		

**Factor 6: Acknowledging the student’s effort [Reconocimiento al esfuerzo del estudiante] (α = 0.948) Mean (*SD*) = 4.51 (0.95)**			

33.	*Por lo que he observado en estos primeros días de clase, cuando nos esforzamos este profesor/a nos lo valora y reconoce, y creo que seguirá siendo así a lo largo del curso.*	0.941F6	885
	From what I have observed on these first days in class, this teacher will value and acknowledge us when we make the effort, and I think this will continue throughout the course.		
34.	*Por lo que he observado en estos primeros días de clase, cuando nos implicamos activamente en el aprendizaje, este profesor/a nos lo valora y reconoce, y creo que seguirá siendo así a lo largo del curso.*	0.863F6	0.744
	From what I have observed on these first days in class, this teacher will value and acknowledge us when we actively engage in learning, and I think this will continue during the academic year.		
35.	*Por lo que he observado en estos primeros días de clase, cuando seguimos sus orientaciones y directrices, este profesor/a nos lo valora y reconoce, y creo que seguirá siendo así a lo largo del curso.*	0.901F6	0.812
	From what I have observed on these first days in class, this teacher will value and acknowledge us when we follow his/her guidance, and I think this will continue throughout the course.		
36.	*Por lo que he observado en estos primeros días de clase, cuando hacemos las cosas bien, este profesor/a nos lo valora y lo reconoce, y creo que seguirá siendo así a lo largo del curso.*	0.903F6	0.815
	From what I have observed on these first days in class, this teacher will value and acknowledge us when we do things well, and I think this will continue throughout the course.		
37.	*Por lo que he observado en estos primeros días de clase, cuando trabajamos por encima de lo exigido, este profesor/a nos lo valora y reconoce, y creo que seguirá siendo así a lo largo del curso.*	0.822F6	0.676
	From what I have observed on these first days in class, this teacher will value and acknowledge us when we work more than what is expected, and I think this will continue throughout the course.		

**Factor 7: Guiding students in their learning [Orientación al studio] (α = 0.935) Mean (*SD*) = 4.24 (1.02)**			

38.	*Desde el principio, el profesor/a nos ha orientado sobre cómo rendir más en esta materia, y creo que seguirá siendo así a lo largo del curso.*	0.879F6	0.773
	Since the beginning, the teacher has guided us about how to perform better in this subject matter, and I think this will continue throughout the course.		
39.	*Desde el principio, el profesor/a nos ha orientado para tener éxito en esta materia, y creo que seguirá siendo así a lo largo del curso.*	0.883F6	0.780
	Since the beginning, the teacher has guided us to be more successful in this subject matter, and I think this will continue throughout the course.		
40.	*Desde el principio, el profesor/a nos ha orientado sobre cómo organizarse y planificarse para obtener buenos resultados en esta materia, y creo que seguirá siendo así a lo largo del curso.*	0.838F6	0.702
	Since the beginning, the teacher has guided us about how to be organized and to plan to obtain good marks in this subject matter, and I think this will continue throughout the course.		
41.	*Desde el principio, el profesor/a nos ha orientado sobre cómo afrontar el aprendizaje de esta materia, y creo que seguirá siendo así a lo largo del curso.*	884F6	0.782
	Since the beginning, the teacher has guided us about how to face learning this subject matter, and I think this will continue throughout the course.		
42.	*Desde el principio, el profesor/a nos ha orientado sobre cómo aprender más y mejor en esta materia, y creo que seguirá siendo así a lo largo del curso.*	0.834F6	0.696
	Since the beginning, the teacher has guided us about how to learn more and better this subject matter, and I think this will continue throughout the course.		

**Factor 8: Self-competency support [Apoyo a la autocompetencia] (α = 0.900) Mean (*SD*) = 4.01 (0.93)**			

43.	*Desde el principio, el profesor/a nos ha tranquilizado al hacernos ver que superar esta asignatura no es difícil.*	0.770F8	0.592
	Since the beginning, the teacher has reassured us by allowing us to see that passing this subject is not difficult.		
44.	*Desde el principio, el profesor/a nos ha transmitido la idea de que todos podemos progresar y tener buenos resultados en esta materia.*	0.871F8	0.758
	Since the beginning, the teacher has transmitted the idea that we can all progress and obtained good outcomes in this subject matter.		
45.	*Desde el principio, el profesor/a nos ha hecho sentir competentes para dominar esta materia.*	0.864F8	0.746
	Since the beginning, the teacher has made us feel competent to master this subject matter.		
46.	*Desde el principio, el profesor/a nos ha hecho sentir bien al hacernos ver que depende de nosotros tener éxito en esta materia.*	800F8	0.639
	Since the beginning, the teacher has made us feel good by allowing us to see that passing this subject depends on us.		
47.	*Desde el principio, el profesor/a nos ha transmitido la idea de que todos estamos capacitados/as para superar esta materia si nos lo proponemos.*	0.716F8	0.512
	Since the beginning, the teacher has transmitted the idea that we are all capable of passing this subject matter if we are determined to.		

**Factor 9: Providing didactic resources [Proporcionar recursos didácticos] (α = 0.878) Mean (*SD*) = 4.61 (0.93)**			

48.	*Los materiales proporcionados por el profesor/a para estudiar y trabajar esta materia son accesibles y fáciles de conseguir.*	683F9	0.466
	The materials provided by the teacher to study and work this subject matter are accessible and easy to obtain.		
49.	*Los materiales proporcionados por el profesor/a para estudiar y trabajar esta materia son claros y comprensibles.*	0.888F9	0.788
	The materials provided by the teacher to study and work this subject matter are clear and understandable.		
50.	*El profesor/a nos ha proporcionado suficientes y variados materiales para estudiar y trabajar esta materia*.	0.784F9	0.615
	The teacher has provided us with enough and varied materials to study and work this subject matter.		
51.	*Los materiales que nos ha proporcionado el profesor/a para estudiar y trabajar esta materia son de calidad.*	0.855F9	0.519
	The materials that the teacher has provided us with to study and work this subject matter are of good quality.		

**Factor 10: Providing Feedback [Proporcionar feedback] (α = 0.861) Mean (*SD*) = 4.46 (0.91)**			

52.	*Por la forma en que está planteada la evaluación, creo que el profesor me supervisará el trabajo para corregir los errores antes de la entrega final.*	0.747F10	0.559
	From the way the evaluation is set out, I think that the teacher will supervise work to correct any mistakes before the final delivered work.		
53.	*El sistema de evaluación otorga mucha importancia al trabajo continuado del estudiante y al feedback del profesor/a.*	0.841F10	0.707
	The evaluation systems attached much importance to students’ constant work and to the teacher’s feedback.		
54.	*Por la forma en que está planteada la evaluación, creo que nos va a ayudar a llevar la asignatura al día y a dosificar el esfuerzo a lo largo del curso.*	0.824F10	0.679
	From the way the evaluation is set out, I think that it will help us to keep the subject up-to-date and to distribute efforts throughout the course.		
55.	*Para asignar la nota final, el profesor/a va a tener en cuenta el esfuerzo y el progreso continuado del alumno/a a lo largo del curso (supervisando tareas, haciendo controles, etc.)*	0.721F10	0.519
	To give the final mark, the teacher will take into account students’ constant efforts and progress throughout the course (supervising tasks, setting tests, etc.).		

**St. Coef., Standardized coefficient.**

#### Reliability Analysis Findings as a Result of the CFA

Cronbach’s alpha internal consistency coefficient for each factor/scale in the instrument (MOCSE-TSQ), was calculated. The Cronbach’s alpha values ranged between 0.95 (maximum) and 0.86 (minimum). See Table 2 for more details.

### Student’s *t*-Test for Gender Differences

Gender differences were examined with the Student’s *t*-test for independent samples (male = 1; female = 2). As noted in Table 3, no remarkable gender differences were generally observed for the construct considered in both measures. The most important difference referred to “Peer support” (*t* = 2.800, sig. = 0.005). All the results are displayed in Table 4.

**TABLE 4:** Student’s *t*-test for gender differences.

Factors/dimensions	Levene test (Sig.)	*t*	*gl*	Sig. (bilateral)	Mean differences
**Teacher supports**					
F1: Content comprehension support	0.076	–1.627	354	0.105	–0.252
F2: Teacher accessibility and closeness	0.097	–2.004	354	0.046	–0.233
F3: Autonomy support	0.055	1.788	354	0.075	0.241
F4: Peer support	0.015	2.800	354	0.005	0.462
F5: Awakening interest in the subject	0.413	–1.771	354	0.077	–0.236
F6: Acknowledging the students’ effort	0.048	–0.589	354	0.556	–0.082
F7: Guiding students in their learning	0.178	–0.490	354	0.625	–0.073
F8: Self-competency support	0.951	–1.292	354	0.197	–0.150
F9: Providing didactic resources	0.477	–0.814	354	0.416	–0.111
F10: Teacher Feed-back	0.872	–0.839	354	0.402	–0.112
**Learning demands**					
F1: Desirability	0.293	0.575	354	0.566	0.053
F2: Feasibility	0.531	–1.526	354	0.128	–0.267

### Pearson’s Bivariate Correlations

Finally, an additional analysis was carried out. A Pearson’s bivariate correlational analysis was performed as an approach to explore the relations between the variables from Stage 1 (demands and supports) and those from Stage 2 (expectancy beliefs and goals adopted by students). The scales from Stage 1 were administered after the first month of class, whereas the scales from Stage 2 were administered 1 month later. Regarding teacher support, positive and significant correlations were generally obtained among teacher support and expectancy of success, process expectancy (the highest values found) and the mastery goals adopted by students, Conversely, negative and significant correlations were obtained between teacher support and avoidance goals. Regarding learning demands, on the one hand, positive and significant correlations were obtained among desirability and expectancy of success, process expectancy (emotional reactions), where the highest value was found (*r* = 0.717, *p* > 0.01), and mastery goals. On the other hand, negative and significant correlations were observed among feasibility and expectancy of success, process expectancy (emotional reactions) and mastery goals. See Table 5 for more details.

**TABLE 5:** Correlation between demands-supports and anticipatory cognitive motivators.

	Intention to learn components (Stage 2)			
Demands (D) and supports (S) (Stage I) (Stage	1.1.Expectancy of success	1.2.Process expectancy	2.1.Mastery goals	2.2.Avoidance goals
D_F1: Desirability	0.278**	0.717**	0.580**	−0.351**
D_F2: Not feasible (too difficult)	−0.239**	−0.174*	–0.120	0.208**
S_F1: Comprehension support	0.214**	0.577**	0.312**	−0.233**
S_F2: Reladness	0.287**	0.552**	0.313**	−0.373**
S_F3: Autonomy support	0.147*	0.388**	0.229**	–0.052
S_F4: Peer support	0.205**	0.068	0.141	–0.088
S_F5: Awakening interest	0.233**	0.484**	0.363**	−0.240**
S_F6: Recognition effort	0.281**	0.492**	0.283**	−0.234**
S_F7: Guiding students	0.278**	0.508**	0.299**	−0.156*
S_F8: Self-competency support.	0.287**	0.353**	0.166*	−0.202**
S_F9: Didactic resources	0.183*	0.462**	0.287**	−0.276**
S_F10: Teacher Feed-back	0.239**	0.500**	0.308**	−0.282**

*p < 0.05; **p < 0.01.

## Discussion

The primary purpose of the present study was to examine the psychometric properties of the “Learning Demands Scale” (MOCSE-LDS) and “Teacher Support Questionnaire” (MOCSE-TSQ) to determine their factorial validity and internal consistency.

The Maximum Likelihood (ML) Robust method of estimation, developed by Satorra and Bentler (1988, 1994), appears to be a good approach when the multivariate normality assumption is violated and/or the sample size is small (Hu et al., 1992; Curran et al., 1996). This study suffers from both problems, which is frequently the case in Social Sciences.

Studies centered on examining the role of learning demands and teacher support in the university context are scarce, but important for improving both teacher practice and students’ motivation, issues traditionally ignored in Higher Education. The results of a meta-analysis conducted by Lei et al. (2018) revealed that teacher support correlated significantly with students’ academic emotions in both no university and the university classroom context.

The EFA conducted of the Learning Demands Scale (MOCSE-LDS) proved the existence of a two-factor structure referring to the constructs of *desirability* and *feasibility* as regards demands. According to the literature based on the Theory of Action Control (Heckhausen and Kuhl, 1985), both constructs are considered crucial in the decision to set a goal intention (Fishbein and Ajzen, 2010). The EFA performed of the Teacher Support Questionnaire (MOCSE-TSQ) revealed the existence of 10 factors or subscales with good psychometric validity and reliability properties.

The CFA conducted on both instruments, using the EQS program, suggested that some minor changes should be made to the initial structure following the recommendations of the Wald and Lagrange test for introducing into or removing parameters. Consequently, the factor structure of both instruments was optimized and fitted the data, which means that the resulting theoretical structure proposed for both the MOCSE-LDS and MOCSE-TSQ was adequate. Moreover, the scales from both instruments showed good internal consistencies, and they all meet the standard of 0.70 recommended by Nunnally and Bernstein (1994). In short, the results confirmed the validity and reliability of both instruments. The data indicated a bidimensional structure of the MOCSE-LDS instrument made up of two factors/subscales named *desirability* and *feasibility*, and a multidimensional structure of the MOCSE-TSQ instrument made up of 10 factors/subscales (Content comprehension support, Teacher accessibility and closeness, Autonomy support, Peer support, Awaken interest in the subject, Acknowledging the student’s effort, Guiding students in their learning, Providing didactic resources, Providing feed-back). Most of the research in the existing literature on this topic have focused on the teaching supports related to the three basic psychological needs (autonomy, competence, relatedness) proposed in the Self-Determination Theory (Deci and Ryan, 2000; Ryan and Deci, 2002). In the present questionnaire, we extended this traditional approach by taking into account additional teacher supports related to instructional and affective-social student needs that previous research has demonstrated as being important for student motivation in Secondary Education (Ahmed et al., 2010; Wentzel et al., 2010; Federici and Skaalvik, 2014a; Federici et al., 2016; Han et al., 2019). We think that such additional supports can also be interesting if they are taken into account in the university context.

To check for gender differences, Student’s *t*-tests for independent samples were performed. Overall, no remarkable gender differences were observed for the construct/dimensions considered in both measures; that is, demands and supports. The most important significant difference referred to the factor “Peer support,” which indicated that males’ perception was much better than females’ perception of this construct. Consequently, it should be taken into account when this construct is measured in the university context.

Finally, regarding the correlational analysis, two things should be highlighted. First, values generally go in the expected direction (e.g., teaching supports showed a positive relation with mastery goals and a negative relation with avoidance goals). This seems to indicate that the two tested instruments (MOCSE-LDS and MOCSE-TSQ) properly measure the constructs considered in the present study. Second, the correlation analysis reveals important associations between the variables from Stage 1 (learning demands and teacher support) and those from Stage 2 (students’ expectancy and goals). Accordingly, an in-depth analysis, following the MOCSE postulates, is suggested in the future at different levels of education and with distinct curricular contents.

In conclusion, the factor validity of both instruments was examined with EFA and CFA based on the MOCSE postulates. The results confirmed the validity and reliability of both instruments which teachers can use to evaluate how their students perceive demands and supports. Likewise, we wish to highlight the importance of having valid assessment instruments like the “Learning Demands Scale” (MOCSE-LDS) and the “Teacher Support Questionnaire” (MOCSE-TSQ) for both applied and research purposes.

### Practical Implications

Both instruments can be useful for researcher and teachers. On the one hand, this study provides researchers with two valid and reliable tools that can contribute to investigate students’ motivation; that is, why a student decides to strive to achieve academic demands/goals or not, and to investigate other related constructs, such as students’ anticipatory cognitive motivators and achievement goals. They were all in accordance with the MOCSE postulates. On the other hand, the two tested instruments can provide teachers and school psychologist with important information to implement preventive or intervention actions that improve students’ intention to learn. Teachers can also use them to evaluate their own teaching and to research their own classrooms. Briefly, the results obtained for the validity and reliability of the two tested instruments are good. Therefore, the application of the instruments MOCSE-LDS and MOCSE-TSQ was satisfactorily supported by the empirical data.

### Limitations and Future Directions

In the present study, the participants were students from Spanish university classes. Although the results obtained in this study are satisfactory, some limitations and suggestions for future research are pointed out. Perhaps the number of the participants in this study is somewhat limited for validating the second instrument (MOCSE-TSQ) composed of 72 items. Further research is needed to investigate whether the validity of the two instruments presented herein is stable for a larger sample, and for other levels of education, and socio-cultural contexts. A computer-based version of both forms is highly recommended to increase accessibility as students can fill in forms outside class. In future studies, more in-depth analyses, like multiple-group structural analyses, could be considered to test the invariance for gender and grade levels. MOCSE-TSQ and MOCSE-LDS will provide important information to understand students’ perceptions of not only the required learning demands in terms of *desirability* and *feasibility*, but also the supports they are expected to be provided with by the teacher during the course for a specific subject matter.

## Data Availability Statement

The raw data supporting the conclusions of this article will be made available by the authors, without undue reservation, to any qualified researcher.

## Ethics Statement

Ethical review and approval was not required for the study on human participants in accordance with the local legislation and institutional requirements. The patients/participants provided their written informed consent to participate in this study.

## Author Contributions

FD-B wrote the first draft of the manuscript and conducted the statistical analyses. AG-A and LA-R reviewed the whole manuscript, checked the references, and made significant contributions. ER-B reviewed the manuscript and contributed to the discussion. All the authors collected the data, reread the manuscript and approved the submitted version.

## Conflict of Interest

The authors declare that the research was conducted in the absence of any commercial or financial relationships that could be construed as a potential conflict of interest.
